# 

**Published:** 1885-04

**Authors:** 


### CONTENTS:

Art. I—Disinfection and Disinfectants.
By Geo. M. Sternberg, M. D., U. S. A. ....529

Art. II—Symptomatic Pathology of the Mouth.

By Elton R. Smilie, M. D., (Continued.) ....540

Art. III—Congenital Alveolar Fissure, With its Accompanying D[illegible]nt[illegible]l Condition,

By Oakley Coles, L. D. S. ....554

Art. IV—Climate Food and Associations in Forming Races. By Jas. Truman, D. D. S. ..559

Art. V—Temporary Teeth. By E. J Lilly, M. D., D. D. S. ....562

Art. VI—Irregularities of the Teeth. By N. Kingsley, D. D. S. ....565

A[illegible] VII—The Missing Incisors in Man, Which are They? ....568

### EDITORIAL, ETC.

[illegible]lar Publication ....571

A [illegible]ission ....571

N[illegible] International Medical Congress ....572

Summer Session of the Dental Department of the University of Maryland ....573

### MONTHLY SUMMARY.

The First Permanent Molar ....574

Removal of the Deciduous Teeth ....575

Death from Nitrous Oxide Gas ....575

